# Targeting γ-secretase triggers the selective enrichment of oligomeric APP-CTFs in brain extracellular vesicles from Alzheimer cell and mouse models

**DOI:** 10.1186/s40035-019-0176-6

**Published:** 2019-12-05

**Authors:** Inger Lauritzen, Anaïs Bécot, Alexandre Bourgeois, Raphaëlle Pardossi-Piquard, Maria-Grazia Biferi, Martine Barkats, Fréderic Checler

**Affiliations:** 1 Institut de Pharmacologie Moléculaire et Cellulaire, CNRS-UMR7275, team labeled «Fondation pour la Recherche Médicale» et «Laboratoire d’excellence Distalz», Université de Nice-Sophia-Antipolis, Sophia-Antipolis, France; 2 Institut-Myologie, Paris, France

**Keywords:** Extracellular vesicles, C99, APP-CTFs, Homo- and hetero-oligomerization, Endosomes, Lysosomes, *trans*-Golgi network, SorLA, γ-Secretase inhibition, Presenilin knockout, Alzheimer’s disease

## Abstract

**Background:**

We recently demonstrated an endolysosomal accumulation of the β-secretase-derived APP C-terminal fragment (CTF) C99 in brains of Alzheimer disease (AD) mouse models. Moreover, we showed that the treatment with the γ-secretase inhibitor (D6) led to further increased endolysosomal APP-CTF levels, but also revealed extracellular APP-CTF-associated immunostaining. We here hypothesized that this latter staining could reflect extracellular vesicle (EV)-associated APP-CTFs and aimed to characterize these γ-secretase inhibitor-induced APP-CTFs.

**Methods:**

EVs were purified from cell media or mouse brains from vehicle- or D6-treated C99 or APP_swedish_ expressing cells/mice and analyzed for APP-CTFs by immunoblot. Combined pharmacological, immunological and genetic approaches (presenilin invalidation and C99 dimerization mutants (GXXXG)) were used to characterize vesicle-containing APP-CTFs. Subcellular APP-CTF localization was determined by immunocytochemistry.

**Results:**

Purified EVs from both AD cell or mouse models were enriched in APP-CTFs as compared to EVs from control cells/brains. Surprisingly, EVs from D6-treated cells not only displayed increased C99 and C99-derived C83 levels but also higher molecular weight (HMW) APP-CTF-immunoreactivities that were hardly detectable in whole cell extracts. Accordingly, the intracellular levels of HMW APP-CTFs were amplified by the exosomal inhibitor GW4869. By combined pharmacological, immunological and genetic approaches, we established that these HMW APP-CTFs correspond to oligomeric APP-CTFs composed of C99 and/or C83. Immunocytochemical analysis showed that monomers were localized mainly to the *trans*-Golgi network, whereas oligomers were confined to endosomes and lysosomes, thus providing an anatomical support for the selective recovery of HMW APP-CTFs in EVs. The D6-induced APP-CTF oligomerization and subcellular mislocalization was indeed due to γ-secretase blockade, since it similarly occurred in presenilin-deficient fibroblasts. Further, our data proposed that besides favoring APP-CTF oligomerization by preventing C99 proteolysis, γ-secretase inhibiton also led to a defective SorLA-mediated retrograde transport of HMW APP-CTFs from endosomal compartments to the TGN.

**Conclusions:**

This is the first study to demonstrate the presence of oligomeric APP-CTFs in AD mouse models, the levels of which are selectively enriched in endolysosomal compartments including exosomes and amplified by γ-secretase inhibition. Future studies should evaluate the putative contribution of these exosome-associated APP-CTFs in AD onset, progression and spreading.

## Background

A growing body of evidence indicate that the β-secretase-derived fragment of βAPP C99 (βCTF) accumulates in brains from various AD mouse models [1] as well as in brains from AD-affected patients [2–5] or in induced pluripotent stem cells (iPSCs) derived from monogenic AD [6, 49]. C99 accumulation was found to mainly occur within endolysosomal compartments and be both a cause and consequence of lysosomal dysfunction [7–9]. Moreover, its accumulation was proposed to be linked to neuronal hyperactivity [10] and LTP alterations [7, 11]. Unexpectedly, our previous work on the 3xTgAD mouse model demonstrated that endolysosomal-associated C99 was only detected with N-terminal-directed and aggregate-specific antibodies but not with C-terminal-directed APP antibodies. This staining was strongly increased in γ-secretase inhibitor treated-mice, in which not only C99 but also C99-derived C83 (hereafter refered to as APP-CTFs) levels were enhanced and those of Aβ reduced, ruling out the possibility that it could be ascribed to Aβ. However, why this endolysosomal APP-CTF immunoreactivity presents an “aggregate-like” conformation remained to be established. Using the AAV-C99 mouse model, expressing C99 in absence of APP overexpression, we delineated the presence of two distinct APP-CTF immunolabelings. Athough we confirmed an endolysosomal-associated staining revealed by N-terminal directed and aggregate specific antibodies in these mice, an additional Golgi-associated staining was recovered by means of antibodies targeting the C-terminal moiety of APP-CTFs, thus proposing the presence of two distinct immunoreactive APP-CTF species. Strikingly, the N-terminal and aggregate-specific antibodies also revealed a diffuse and extracellular immunostaining in both AAV-C99 and 3xTgAD mice which was also strongly increased in γ-secretase inhibitor-treated animals. Since APP-CTFs are membrane-embedded, and thus, should not be recovered in the extracellular space, we postulated that this staining could correspond to exosome-associated APP-CTFs, which would then be particularly important in γ-secretase-treated animals. Amyloidogenic APP processing is thought to mainly occur in the endosomal system [12, 13], in which exosomes are formed [14], but previous studies have shown that in AD, the exosomal content of Aβ is actually quite low and accounts for less than 1% of the total Aβ content [15]. Conversely, a growing body of evidence indicates that the membrane-bound APP-CTFs, including the C99 fragment, the direct precursor of Aβ [16], are abundant in extracellular vesicles [17, 18], but little is known about the mechanisms underlying their exosomal accumulation, role, fate and subsequent functional consequences. In the present work, we confirmed the strong abundance of APP-CTFs in purified extracellular vesicles (EVs) from various cellular and mouse AD models and found that the treatment with the γ-secretase inhibitor D6 led to further increased levels of C99 and C99-derived C83. Surprisingly, D6 treatment also unraveled the occurence of several high molecular weight (HMW) APP-CTFs, the levels of which were hardly detectable in cell or brain homogenates. Previous in vitro data suggest that C99 can exist not only as monomers but also as dimers and that dimerization is favored by three repeats of the glycine-xxx-glycine (GXXXG) motif present in the juxtamembrane and transmembrane regions [19]. Thus, we questioned whether these HMW APP-CTFs could be dimeric/oligomeric APP-CTFs and used pharmacological and immunological approaches, as well as GXXXG dimerization mutants [19] to determine the exact nature of those APP-CTFs. Taken together, we establish that these HMW APP-CTFs indeed correspond to dimeric/oligomeric APP-CTFs, including not only C99 homodimers but also C83 homodimers and C99/C83 oligomers. Furthermore, our data showed that the selective recovery of dimeric/oligomeric APP-CTFs in EVs is linked to their intracellular mislocalization to the endolysosomal network due partly to a defective SorLA-mediated retrograde sorting of dimeric APP-CTFs to the TGN.

## Materials and methods

### Animals, viral infection and ELND006 treatment

3xTgAD (harboring βAPP_swe_, and Tau_P301L_ transgenes on a PS1_M146V_ background) and non-transgenic (wild-type) mice [20] were generated from breeding pairs provided by Dr. LaFerla (Irvine, USA). AAV-10 production and AAV-mediated in vivo delivery was described previously [7]. Briefly, 1 day old C57BL6 mice (Janvier Labs., France) were injected unilaterally with 4 μl of AAV virus, AAV-C99 or AAV-free (under control of the synapsin I promoter) (5.5 × 10^12^ vg/ml (viral genomes *per* ml)) into the left lateral ventricle and mice were analyzed at 2 months post-AAV delivery. 3xTgAD and wild-type mice, as well as AAV-infected mice were treated daily for 15 days with the γ-secretase inhibitor ELND006, referred to as D6 hereafter (30 mg/kg, Elan Pharmaceuticals, San Francisco) or with vehicle alone (methylcellulose/polysorbate 80, Sigma) via oral gavage, as described [21]. For the purification of brain EVs (see below), mice were anesthetized by intraperitoneal injection of Ketamine (100 mg/kg) and Xylazine (24 mg/kg) and intracardiacally perfused with PBS before sacrifice. For immunohistochemistry, mice were perfused intracardically with PBS followed by paraformaldehyde 4% before collecting the brains. All animals were housed with a 12:12 h light/dark cycle and were given free access to food and water and experimental procedures were in accordance with the European Communities Council Directive of 24 November 1986 (86/609/EEC) and local French legislation.

### Plasmid constructs

The pcDNA_3_ SPC99_G33L_ construct was generated using the QuickChange II Site-Directed Mutagenesis Kit (Agilent Technologies) with pcDNA_3_ SPC99 previously described (flammang 2012) and appropriated primers: 5′- AAG GCG CAA TCA TTC TAC TCA TGG TGG GCG GTG - 3′ and 5′- CAC CGC CCA CCA TGA GTA GAA TGA TTG CGC CTT - 3′. The pcDNA_3_ SPC99_G29L/G33L_ plasmid was obtained using the same protocol with the pcDNA_3_ SPC99_G33L_ previously generated and the following primers: 5′- GGG TTC AAA CAA ACT CGC AAT CAT TCT ACT C - 3′ and 5′ - GAG TAG AAT GAT TGC GAG TTT GTT TGA ACC C - 3′). The doxycyclin-inductible pSBtet SPC99 construct used for stable cell line generation was obtained as following. First, the SPC99 fragment was amplified by PCR from the pcDNA3 SPC99 using the following primers (5′– ATA TTA GGC CTC TGA GGC CCC ACC ATG CTG CCC GGT TTG GCA C – 3′ and 5′– GAT GGC CTG ACA GGC CCT AGT TCT GCA TCT GCT CAA AGA ACT TG TAG GTT – 3′) to introduce the SfiI restriction site at both 5′ and 3′ end of fragment. The resulting product was then digested by SfiI and subcloned into the pSBtet vector. All constructs were verified by sequencing. Rab5-GFP, Rab7-GFP and Lamp1-GFP were from Addgene and the SorLA_myc_ construct was a kind gift from Peter St-George-Hyslop.

### Cell culture and treatment

Human neuroblastoma (SH-SY5Y, ATTC or SH-SY5Y-APPswe [22]), human embryonic kidney cells (HEK293, ATTC), human epitheloid cervix carcinoma (HeLa, ATCC) and mouse embryonic fibroblasts (MEFs, wildtype or devoid of PS1 and PS2, PS1/2^−/−^) [23] were cultured in Dulbecco’s modified Eagle’s medium supplemented with 10% fetal calf serum, penicillin (100 U/ml) and streptomycin (50 μg/ml) purchased from Life Technologies (CA, USA) at 37 °C/5% CO_2_. Transient transfections of cells were carried out using Lipofectamine 2000 (Life Technologies) for SH-5YSY and MEFs and JetPrime (Polyplus transfection) for HEK293 and HeLa cells, according to the manufacturer’s instructions, and cells were recovered 24–36 h post-transfection. For immunofluorescence analysis, some cells were co-transfected with C99 or C99^G29L/G33L^ and plasmids expressing intracellular organelle-specific proteins (Rab5-GFP, Rab7-GFP and Lamp1-GFP, Addgene). Stable inducible HEK293 and SH-SY5Y cell lines were obtained by co-transfection of the Sleeping Beauty inductible vector (pSBtet SPC99) and the transposase SB100 using JetPrime and Lipofectamine 2000, as described above, and using puromycin selection. For stable cell lines, protein expression was induced by the addition of doxycyclin (10 μg/ml final concentration, Sigma) to the cells 24 h before cell treatments: D6, 1 μM (Elan Pharm.), GI254023X 5 μM (Sigma), or GW4869 10 μM (Sigma), which were added to OptiMEM. Cells for western blot analysis were lysed in RIPA buffer and sonicated. For immunocytochemistry, SH-SY5Y cells were grown on poly-D-lysine coated coverslips and treated as indicated above.

### Immunostaining of tissue and cells

Paraformaldehyde-fixed brains (see above) were embedded in paraffin and cut on a microtome in 8 μm thick sections (Thermoscientific, France) or cut directly on a vibratome in 50 μm thick sections (Thermoscientific, France). Brain sections were treated with formic acid 50%/5 min and saturated with 5% BSA/0.1% Triton and then incubated at 4 °C overnight with primary antibodies (NU1 [24], 1:1000) or 4G8 (Covance, 1:2000), followed by Alexa Fluor-488 conjugated anti-mouse (Molecular Probes, 1:1000) and DAPI (Roche, 1:20000) or HRP-conjugated antibodies (Jackson ImmunoResearch, 1:1000) and DAB substrate (Vector). For co-labeling with Iba1 or GFAP vibratome sections were processed as described and incubated at 4 °C overnight with 4G8 (Covance, 1:2000) and Iba1 (Wako, 1:2000) or anti-GFAP (Abcam, 1:2000) followed by Alexa Fluor-488 and Alexa Fluor-594 conjugated antibodies (Molecular Probes, 1:1000). Cells in culture were paraformaldehyde fixed, permeabilized with 0.1% Triton-X 100 for 10 min, saturated in 5% BSA/0.1% Tween20, probed 1 h with primary antibodies (α-APPct, rabbit polyclonal, gift from Paul Fraser, 1:5000 and in some cases with α-TGN46 (Serotec, AHP500G, 1:1000) and detected with Alexa Fluor-488 or Alexa Fluor-590 conjugated antibodies (Molecular Probes, 1:1000). Confocal images were acquired using a Zeiss SP5 confocal microscope for cells and an Olympus Fluoview10 confocal microscope for tissue sections. Quantification of APP-CTF localization was performed in a double-blind manner. A total number of 400–500 cells were counted for each condition in 3 independent experiments. For each cell, APP-CTF localization was determined as “Golgi/ER” pattern or “endosome/lysosome” pattern.

### Purification of EVs

Cells at about 80% of confluence (minimum 3 × 150 mm dishes per condition) were rinsed twice with PBS and cultivated for another 20-22 h in OptiMEM for exosomal secretion. When using inducible cell lines, protein expression was induced by the addition of doxycycline (10 μg/ml) 1 day before the change to OptiMEM, which also contained doxycycline. Media were harvested and spun at 2000 g for 20 min and filtered through a 0.22 μm filter. The supernatant was then sequentially centrifuged at 10000 g for 30 min and 100,000 g for 125 min. The pellet was washed in ice-cold PBS and centrifuged for another 120 min. The final pellet (P100) was resuspended in PBS. For western blot, RIPA buffer 5X× was added and EVs were sonicated in an ultrason bath for 15 min. EVs isolated from the brain extracelullar space were purified according to the protocol described by Perez-Gonzalez et al. [25] with minor modifications. As described above, mice were perfused intracardically with ice-cold PBS to remove blood extracellular vesicles. Then, hemibrains were cut in 4–5 pieces and enzymatically and mechanically dissociated using “the adult brain dissociation kit” (Miltenyi Biotec) and a GentleMACS Dissociator (Miltenyi Biotec). 10 ml of PBS was added to the cell suspension, which was then filtered through a 70 μm Smartstrainer. The suspension was then spun at 300 g for 10 min and the supernatant was used for exosome purification and the cell pellet for analysis by western blot. The supernatant was sequentially centrifuged at 2000 g/20 min, 10,000 g/30 min and 100,000 g for 90 min. The pellet was then washed in PBS, centrifuged another 90 min and the pellet was resuspended in 2 ml sucrose 0.95 M and introduced into a sucrose gradient (2, 1.65, 1.3, 0.95, 0.6 and 0.25 M, 2 ml each). The sucrose gradient was centrifuged at 200000 g/16 h. 1 ml fractions were collected from the top of the gradient and fractions flanking the interphase separating 2 neighboring sucrose layers were pooled together (a-top 1 ml fraction, b-2 ml, c-2 ml, d = 2 ml, e = 2 ml, f = 2 ml, and g = bottom 1 ml fractions) diluted with 10 ml PBS and centrifuged at 100000 g for 90 min. Accordingly to the original protocol, our preliminary experiments using wild-type mouse brains showed that 3 fractions (b, c and d) contain extracellular vesicles, thus in all further experiments, these fractions were pooled, centrifuged at 100000 g and resuspended in 200 μl of PBS. Then 20 μl was recovered for Nanoparticle tracking analysis (NTA) or electron microscopy (EM), and the remaining extracellular vesicles were lysed by the addition of RIPA5X, proteinase inhibitors and ultrasonicated. 30 μl of the lysate (corresponding to about 10% of the total volume) was used for each lane on the immuno blots.

### Electron microscopy and nanoparticle tracking analysis (NTA)

For EM, a drop of EVs was added to a freshly ionized 300 mesh formvar/carbon coated grid and incubated for 5 min to allow adherence of the EVs to the grid. The grid was then washed through 5–7 puddles of ddH2O; and negatively stained in 2% aqueous uranyl acetate for 30 s, then visualized using a JEM 1400 electron microscope operating at 100 kV equipped with a Morada SIS camera. For NTA, vesicles diluted to the appropriate concentrations to permit counting in the range of 1 × 10^8^-1 × 10^10^ particles/ml were injected into the Nanosight apparatus equipped with a syringe pump (Nanosight NS300, Malvern, France). The EVs were visualized by their scattering with a CCD video camera. Pump speed was set to 50, camera level at 16 and the detection threshold at 3. Particle size and number was determined from 3 (60 s) videos, and averaged using the NTA software.

### Western blot analysis

Cell or brain homogenates (10 μg) and EV proteins (30 μl) were separated by Bio-Rad 12% stain-free™ TGX FastCast™ acrylamide gels or 16% tris-tricine gels (for the detection of APP-CTFs). Bio-Rad gels were photoactivated for the visualization of proteins and used for quantification of exosomal proteins (indicated in the Figures as protein stain) before being electrophoretically transferred to nitrocellulose membranes using the Bio-Rad Trans-Blot® Turbo™ Transfer System. Tris-tricine gels were directly transferred to nitrocellulose membranes and boiled in PBS before saturation with milk. Membranes were blotted with the following antibodies: α-APPcter (Rabbit polyclonal, gift from Paul Fraser, 1:1000), WO-2 (Sigma, 1:1000), α-Alix (Santa Cruz, 1A2A, sc-53,540, 1:1000), α-Hsc70 (Santa Cruz, B-6, sc-7298, 1:1000), α-Tsg101 (Santa Cruz, C-2, sc-7964, 1:1000), α-Flotilin-2 (Santa Cruz, H-90, sc-25,507, 1:500), α-Calnexin (Santa Cruz, AF18, sc-23,954, 1:500) or α-Actin (Sigma, 1:5000). After probing with primary antibodies, immunological complexes were revealed with HRP-conjugated antibodies (Jackson ImmunoResearch, 1:10000) followed by electrochemiluminescence (Westernbright™ Sirius™ and Quantum™ chemiluminescent HRP substrate, Advansta, France). Immunoblots for APP-CTFs were exposed for various times to ensure the detection of all APP-CTF immunoreactivities and the non-saturation of highly expressed APP-CTFs. Peak height of signal intensities from protein bands were quantified with ImageJ software.

### Co-immunoprecipitation experiments

Stable inducible C99-expressing HEK293 cells were transiently transfected with a myc-tagged SorLA construct using Lipofectamine 2000 and treated or not with D6, as described above. Cells were lysed in RIPA buffer and 500 μg lysates were precleared with 20 μl Protein A Sepharose (GE Healthcare) for 2 h at 4 °C. Then the supernatant was incubated for 1 h with 1 μl α-APPct antibody before adding 30 μl Protein A Sepharose beads. After several washes in RIPA buffer, the beads were resupended in SDS sample buffer (2×) and incubated for 5 min at 95 °C. 20 μl of the sample was loaded on either 8% or 12% Bio-Rad stain-free™ TGX FastCast™ acrylamide gels and subjected to western blot analysis using either an anti-myc antibody (clone 9E10, Sigma, 1:5000) or α-APPct (1:1000).

### Statistical analysis

All quantitative data were subjected to non-parametric tests, the Mann-Whithney U test for single comparison and one-way ANOVA analysis for multiple comparisons followed by Dunnets post-hoc analysis using GraphPad Prism 7. Data are represented as means ± SEM. Statistical significance is represented by asterix * *p* < 0.05, **p < 0.01 and ***p < 0.001.

## Results

### Purified EVs from C99- or APP- expressing cells are enriched in APP-CTFs and contain high molecular weight APP-CTFs, the levels of which are increased by γ-secretase inhibition

**Fig. 1:**
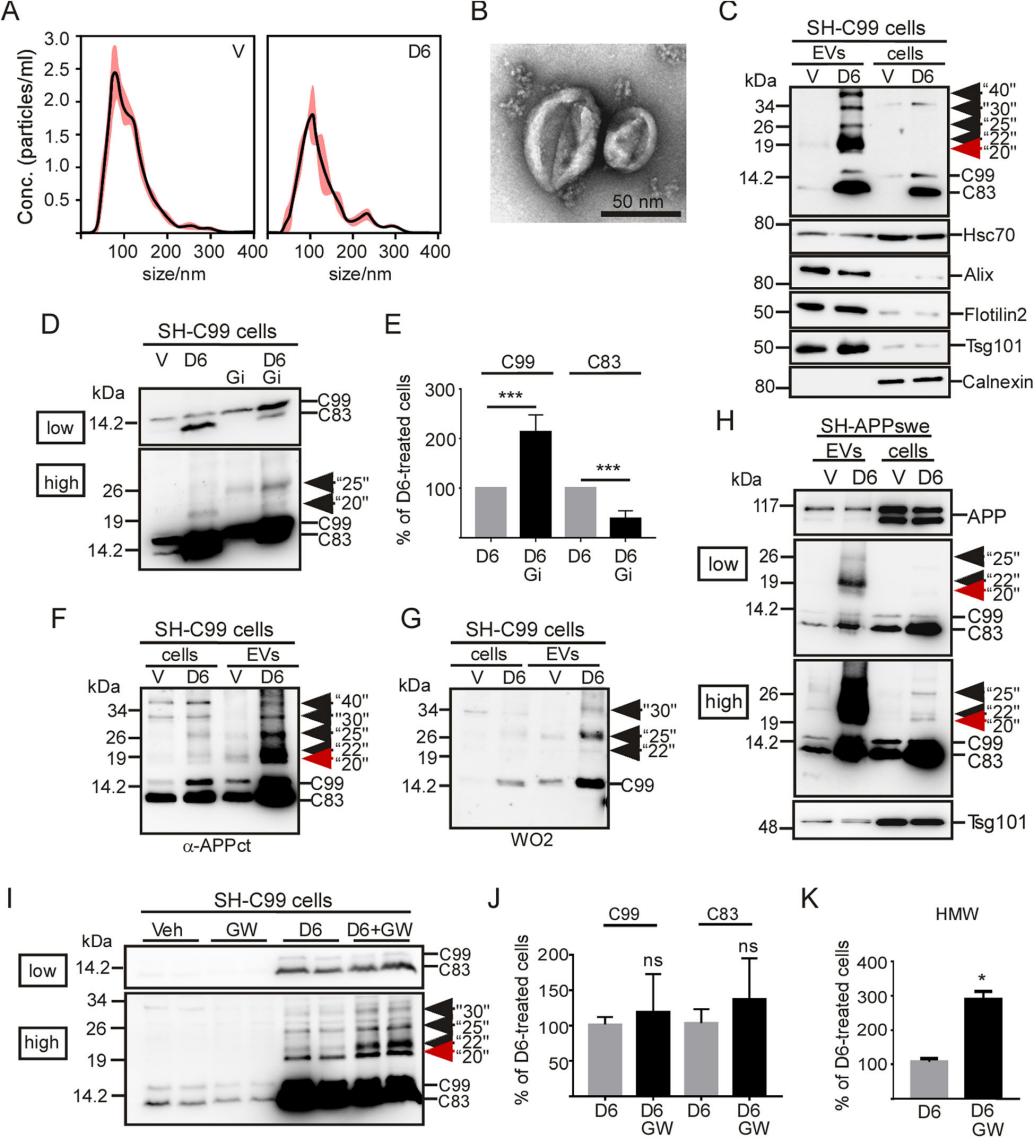
EVs from γ-secretase inhibitor-treated C99 expressing SH-5YSY are enriched in high molecular weight APP-CTFs. **a-c** EVs were purified from media of vehicle- or D6-treated SH-C99 cells and validated by NTA (**a**) and electron microscopy (EM) (**b**) and immunoblot analysis of exosomal markers (**c**). **a-b** Both NTA and EM revealed the presence of vesicles having a mean size around 100 nm in both conditions. **c** Immunoblots showed the presence of the exosomal markers Hsc70, Alix, Flotilin2 and Tsg101 and the absence of the reticular marker Calnexin. **c** The same immunoblots were used to detect APP-CTFs. Arrows point to distinct high molecular weight (HMW) immunoreactivities revealed with α-APPct. **d-e** SH-C99 cells were treated or not with D6 and with or without the α-secretase inhibitor GI254023X (Gi), and intracellular APP-CTF levels were analyzed by immunoblot. The same blot is illustrated at low (low) and high (high) exposure. Bars in (**e**) correspond to the levels of C99 and C83, as normalized to the levels in D6-treated cells. Data represent means ± SEM, ***p < 000.1 as analyzed by the Mann Whitney U-test. f-g APP-CTFs were detected in EVs or cell lysates (cells) using the α-APPct antibody (**f**) or the N-terminal directed antibody WO-2 (**g**). Arrows point to HMW immunoreactivities. Note that some of the immunoreactivities revealed with α-APPct (particularly at the red arrow) were not detected with WO-2. **h** APP-CTFs were detected by immunoblot in EVs or whole lysates (cells) of vehicle- or D6-treated SH-SY5Y-APPswe cells using α-APPct. Arrows point to HMW-APP-CTFs. Low and high correspond to low or high exposures. **i-k** SH-C99 cells treated or not with D6 and with or without GW4869 (GW) were analyzed for APP-CTF levels by immunoblot using the α-APPct antibody. The same blot is illustrated at low (low) and high (high) exposure. Arrows point to HMW. Bars in (**j** and **k**) correspond to the quantification of C99 and C83 (**j**) or HMW immunoreactivities (**k**), as normalized to the levels in D6-treated cells. Data represent means ± SEM, *p < 0.05 as analyzed by the Mann Whitney U-test

Our previous data obtained from immunostaining on brains from 3xTgAD and C99-expressing mice proposed the presence of extracellular vesicle-associated APP-CTFs, the levels of which should be increased in γ-secretase inhibitor treated mice [7]. In order to confirm this hypothesis and to delineate the influence of γ-secretase inhibition on EV- associated APP-CTFs, we first purified EVs secreted from cell media of vehicle- or γ-secretase inhibitor-treated (D6) C99-expressing SH-SY5Y neuroblastoma (SH-C99) (Fig. 1). Vesicle purification was firstly verified by nanoparticle tracking analysis (NTA) (Fig. 1a) and electron microscopy (Fig. 1b) revealing the presence of cupformed vesicles with a mean diameter around 100 nm, as expected for exosomes [26]. Furthermore, immunoblot analysis showed the presence of several exosomal markers (Hsc70, Alix, flotilin-2 and Tsg101) and the total lack of the reticular marker calnexin (Fig. 1c), proposing that these EVs could mainly correspond to the endosome-originating exosomes [27]. Immunoblot analysis also revealed the presence of C99 and C83 in both EVs and whole lysates from SH-C99 cells and showed a strong increase in both of these APP-CTFs upon D6 treatment (Fig. 1c). Despite the fact that these cells specifically expressed C99, the levels of C83 were higher than C99, and this was particularly the case in EVs. In agreement with previous works [28, 29], we confirmed that this C83 was derived from α-secretase-mediated cleavage of C99, since the α-secretase inhibitor GI254023X (Gi) led to a virtually complete decrease of D6-induced C83 with a concomitant increase in C99 levels (Fig. 1d, e). Besides the strong effect of γ-secretase-inhibition on the levels of C99 and C83, more surprisingly, analysis of EV contents also unraveled the presence of various high molecular weight (HMW) APP-CTF immunoreactivities (around 20-, 22-, 25-, 30- and 40 kDa, respectively) (Fig. 1c). These APP-CTFs were observed in vesicles from D6-treated SH-C99 cells but not from mock-transfected cells (not shown), indicating that they were directly linked to C99. The levels of HMW APP-CTFs were very high in EVs, while they were hardly detectable in cell homogenates, in which C99 and C83 were the predominant species (Fig. 1c, f). Nevertheless, when higher protein loads were analyzed (Fig. 1f), HMW APP-CTFs could also be detected in D6-treated cell lysates, indicating that the formation of these APP-CTFs, at least partly, originate from intracellular compartments. The presence of HMW APP-CTF species of different sizes suggested that they could correspond to oligomeric APP-CTFs of different compositions. In agreement with this hypothesis, the treatment with the α-secretase inhibitor Gi reduced the levels of the HMW band around 20 kDa (thus probably composed of C83) and concomitantly increased the band around 25 kDa (thus probably containing C99) (Fig. 1d). Furthermore, also in EVs, only part of the APP-CTF immunoreactivities was detected with the N-terminal directed antibody WO-2 (Fig. 1g), which recognizes C99 but not C83 (compare labeling in Fig. 1f and Fig. 1g). Finaly, to confirm the presence of HMW APP-CTFs in cells, in which C99 is not directly expressed but produced from APP, we also purified EVs secreted from SH-SY5Y cells stably expressing APPswe (Fig. 1h). Also in these cells, D6-treatment unraveled EVs containing not only C99 and C83 monomers but also HMW APP-CTFs, which levels again were low and almost undetectable in homogenates of cells.

### The treatment of SH-C99 cells with the inhibitor of exosomal production and secretion GW4869 leads to increased levels of intracellular high molecular weight APP-CTFs

The above described data indicated a high content of APP-CTFs in EVs and suggested that many of these EVs could correspond to exosomes. Exosome production is dependent on the conversion of sphingomyelin into ceramide by neutral sphingomyelinase (nSMase) [30]. Thus, to strengthen our hypothesis, we assumed that GW4869 an inhibitor of nSMases should affect cellular HMW APP-CTF levels and immunological profile and mimic those observed in EVs. Indeed, the treatment of SH-C99 cells with GW4869 (GW) led to increased intracellular levels of D6-induced HMW APP-CTFs (Fig. 1i, k), the electrophoretic profile of which resembled those detected in EVs, while monomeric APP-CTF levels remained almost unaffected (Fig. 1i, j). This observation supported our previous statement that HMW APP-CTFs at least partly, could originate from intracellular compartments.

### Purified EVs from C99-expressing animals are enriched in APP-CTFs and contain high molecular weight APP-CTFs, the levels of which are increased by γ-secretase inhibition

**Fig. 2:**
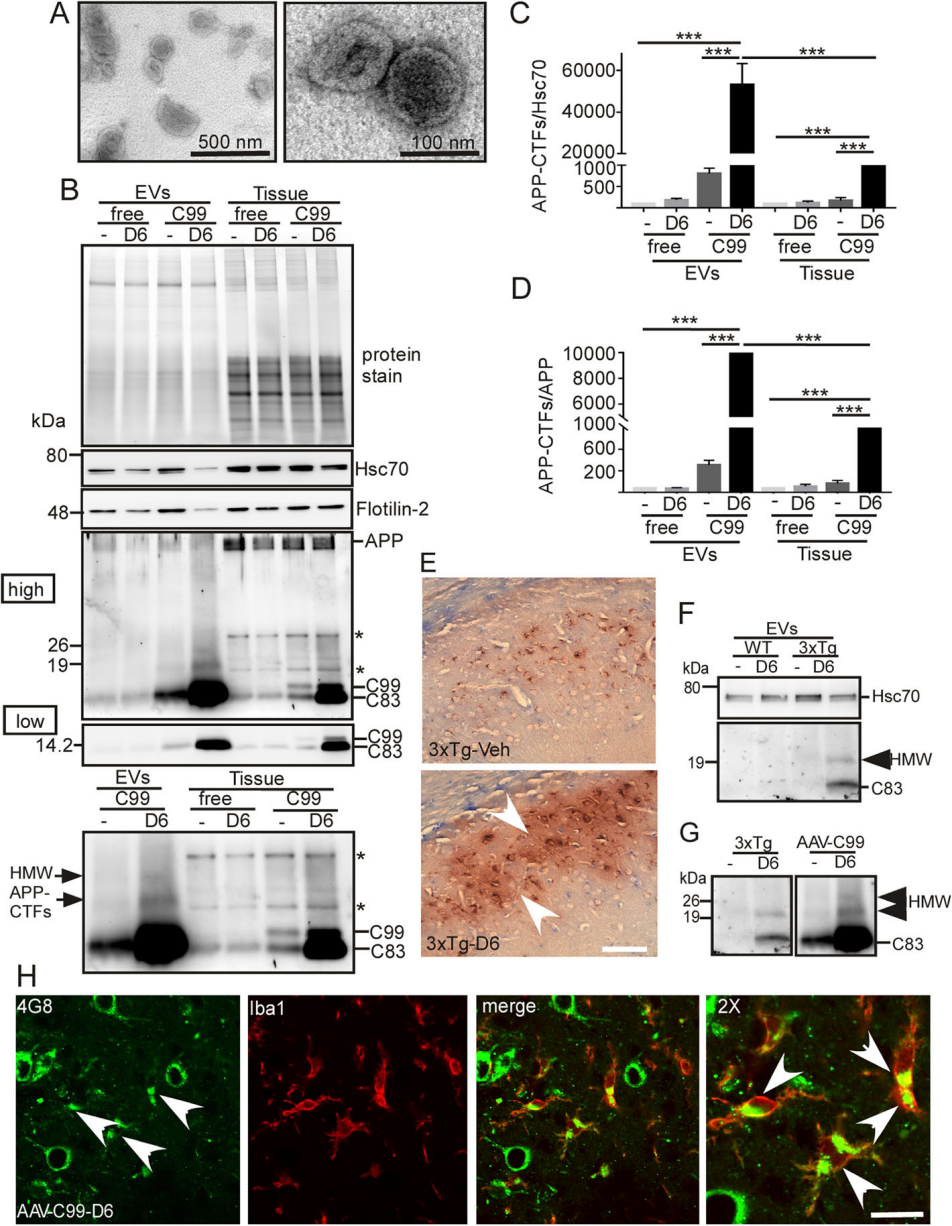
EVs from γ-secretase inhibitor-treated AAV-C99 and 3xTgAD mice are enriched in APP-CTFs including high molecular weight APP-CTFs. **a**-**d** Brain EVs from 2 month-old vehicle- or D6-treated AAV-free or AAV-C99 mice were validated by electron microscopy (**a**) and immunoblot for the detection of the exosomal markers Hsc70 and Flotilin2 (**b**). **a** Ultramicrophotographs of vesicles purified from D6-treated AAV-C99 brain showed the presence of mainly vesicles with sizes around 100 nm. Bar scales correspond to 500 nm and 100 nm, respectively. **b-d** APP-CTFs in EVs and whole brain (tissue) were detected by immunoblot analysis. Proteins were stained by photoactivation using the Bio-Rad prestain method (protein stain) and immunoblots were used for the detection of APP-CTFs and exosomal markers (Hsc70 and Flotilin-2). The α-APPct antibody detected some non-specific immunoreactivites in tissue extracts (asterix). Low and high correspond to low or high exposures of the same immnoblot. The lower blot illustrates the presence of HMW APP-CTFs at higher magnification. Bars in **c-d** correspond to the levels of APP-CTFs in EVs or whole brain (Tissue) that were normalized to either endogenous Hsc70 (**c**) or full-length APP (**d**). Data represent means ± SEM, *p < 0.001 as analyzed by 2-way ANOVA followed by Dunetts posthoc analysis. **e** Brain sections of 5 month-old 3xTgAD vehicle- or D6-treated mouse were immunostained with 4G8 and microphotographs illustrates the staining at the level of the subiculum. Note the strong extracellular staining in the D6-treated mouse arrow heads. **f-g**, EVs were purified from hippocampi of vehicle- or D6-treated Wild-type (WT) and 3xTgAD mice and used for immunoblot analysis of APP-CTFs. Blots in (**g**) allow the comparison of the sizes of HMW APP-CTFs detected in 3xTgAD and AAV-C99 mouse brain vesicles. **h** Co-immunostaining of APP-CTFs with 4G8 (green) and α-Iba1 (red) on brain sections of D6-treated AAV-C99 animals. Arrows in the merged image point to APP-CTF positive microglia. Bar in e and h corresponds to 100 μm and 10 μm, respectively

Next, we seeked to confirm the adequation between the above described observations from in vitro cultured cells and our previous immunohistochemical data on AAV-C99 mice revealing D6-induced increase in extracellular APP-CTF-like imunoreactivities [7]. Brain EVs from γ-secretase inhibitor- (D6) or vehicle-treated AAV-free or AAV-C99 mice [7] (Fig. 2a-d) were purified according to a recently described protocol [25]. As seen for EVs secreted from cultured cells, electron microscopy (Fig. 2a) and NTA (not shown) confirmed the presence of appropriate sized and shaped vesicles for exosomes [26]. Furthermore, immunoblot analysis revealed the presence of several exosomal markers including Hsc70 and Flotilin-2 (Fig. 2b). In AAV-free mice, immunoblot showed the presence of only endogenous C83 in both EVs and brain lysates (Fig. 2b), while both C99 and C83 were detected in tissue lysates of the AAV-C99 mice. As observed in cells, the levels of C83 were higher in C99-expressing mice than in the AAV-free mice, indicating that most of C83 directly derives from C99 in AAV-C99 mice (Fig. 2b). Moreover, as described for EVs from cultured cells, C83 was more abundant than C99 in EVs from both control and D6-treated AAV-C99 mice (Fig. 2b), suggesting that virtually full α-secretase-mediated conversion of C99 into C83 occurs within EVs. D6-treatment led to drastic enhancement of APP-CTF levels in EVs (Fig. 2b). Indeed, the APP-CTF/Hsc70 ratios in EVs from AAV-C99-vehicle and AAV-C99-D6 mice were more than 60 and 500 times higher respectively, than the ratio in AAV-free EVs (Fig. 2c). In comparison, in whole brain homogenates, the APP-CTF/Hsc70 ratios were only 2 times higher in AAV-C99-vehicle mice and 75 times higher in AAV-C99-D6 mice, as compared to AAV-free mice (Fig. 2c, f (7,24) = 97.83, *p* < 0,001; one-way ANOVA). Similarly, the APP-CTF/APP ratios were about 6 and 113 times higher in EVs from AAV-C99 vehicle-treated mice and D6-treated mice, respectively, than in EVs from AAV-free mice (Fig. 2d). Again, in whole brain homogenates, APP-CTF/APP ratios were only 2 and 30 times higher in vehicle- and D6-treated AAV-C99 mice, respectively, than in AAV-free mice (Fig. 2d, f (7,24) = 24.77, one-way ANOVA)). Overall, this set of data indicated that D6 treatment led to the secretion of strongly APP-CTF-enriched EVs in AAV-C99 mice. Moreover, as observed in SH-C99 cells, immunoblot also revealed the presence of HMW APP-CTF immunoreactivities in EVs purified from AAV-C99 mice, the levels of which were also further increased in EVs from D6-treated mice (Fig. 2b). The HMW species were totally absent in AAV-free mice and thus directly linked to C99 or its derived products such as C83. These findings were also confirmed in EVs purified from 3xTgAD mouse brains in which C99 is produced from overexpressed APP_swedish_ (Fig. 2e-g). Indeed, these mice also displayed extracellular APP-CTF associated staining that was restricted to the subiculum of D6-treated animals, the area accumulating C99 (Fig. 2e). Moreover, biochemical analysis of EVs purified from hippocampi revealed the presence of both monomeric (C83) and at least one HMW APP-CTF in D6-treated 3xTgAD mice. This HMW APP-CTF had a size similar (about 20 kDa) to the most abundant HMW APP-CTFs observed in AAV-C99 mouse brains and probably corresponds to C83 homodimers (Fig. 2g). It is well known that once secreted, EVs can be taken up by neighbouring cells. Thus, in order to determine the fate of the neuronal-derived APP-CTF bearing EVs in our mouse models, we performed co-immunostaining on AAV-C99 mouse brain sections to detect APP-CTFs (with the APP antibody 4G8) and to depict microglial (Iba1 antibody) and astroglial (GFAP antibody) cells. Indeed, despite the fact that in our mouse model C99 expression is driven by the neuronal promoter synapsin, this analysis revealed the presence of multiple 4G8-immunostained Iba1-positive cells (Fig. 2h), suggesting that many of these APP-CTF bearing EVs were taken up by microglial cells. 4G8 immunostaining was not detected in GFAP positive cells (not shown), suggesting a lower uptake of these EVs in astroglia.

### The dimerization C99 mutant favors the formation of high molecular weight APP-CTFs resembling those observed in D6-treated cells

**Fig. 3:**
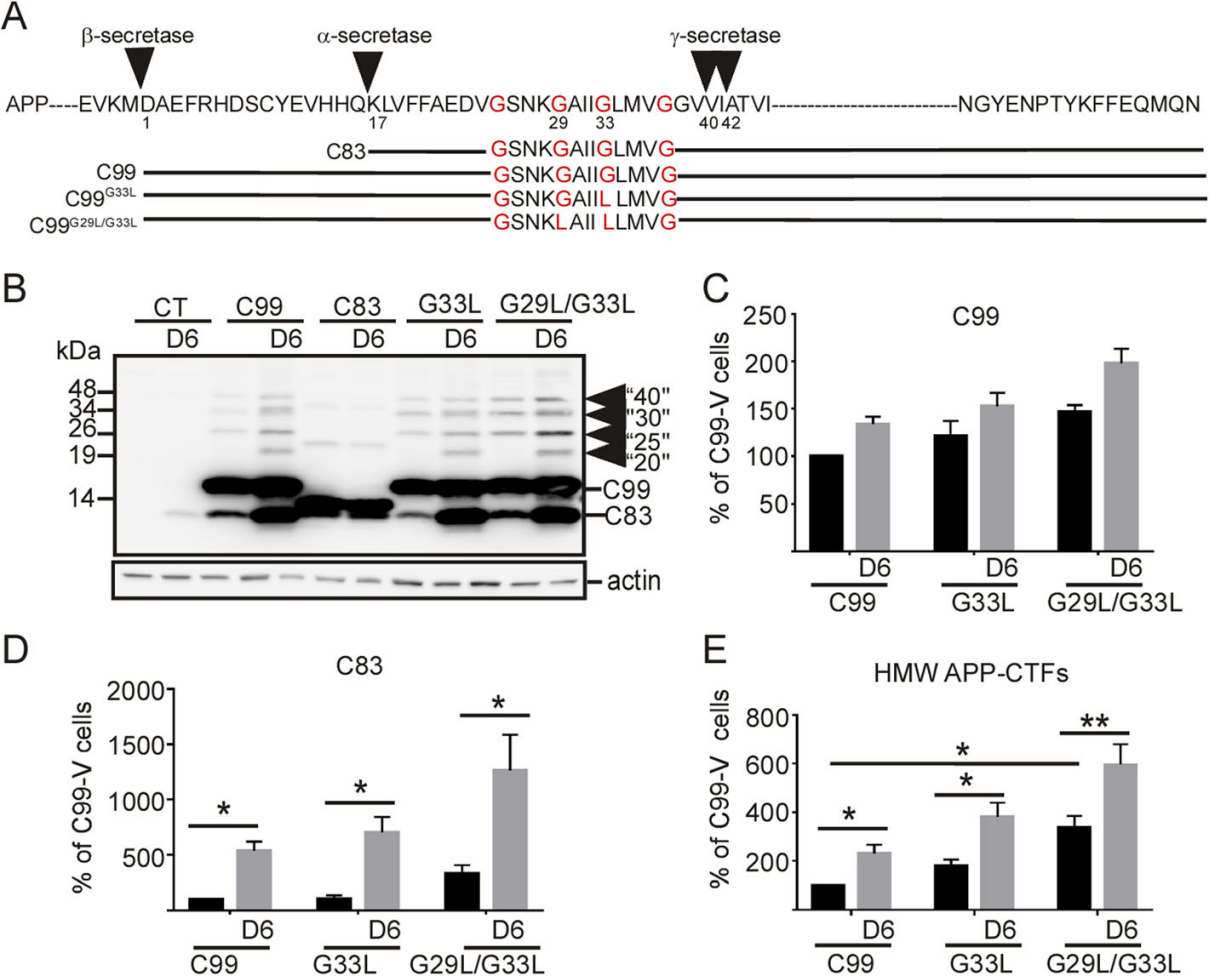
Mutations in the GXXXG motif in the transmembrane domain of C99 promote the formation of HMW APP-CTFs resembling those induced by D6-treatment. **a** shows a schematic representation of APP, C83, C99 and C99 dimerization mutant sequences used in this study. Glycines (G) substituted by leucines (L) are indicated in red. **b**-**e** HEK293 cells were transfected with empty vector (CT), C99, C83, C99^G33L^ or C99^G29L/G33L^_,_ and were treated with or without D6. Arrows point to the high molecular weight (HMW) APP-CTF immunoreactivities. Bars in (**c-e**) correspond to the levels of C99 (**c**), C83 (**d**) and HMW APP-CTF (**e**) immunoreactivities, which in each case were normalized to the levels in vehicle-treated C99 expressing cells. Data represent means ± SEM, *p < 0.05, **p < 0.01 as analyzed by 2-way ANOVA followed by Dunetts posthoc analysis

The data obtained with the α-secretase inhibitor and immunological studies suggested that the HMW-APP-CTFs could correspond to oligomeric APP-CTFs. To further confirm this postulate, we generated two GXXXG C99 mutants, C99^G33L^ and C99^G29L/G33L^ previously proposed to promote homodimerization [19] (Fig. 3a) and expressed them into HEK293 cells for high-yield gene expression. Indeed, we observed that the expression of C99^G29L/G33L^ led to the detection of not only monomeric C99 but also of HMW APP-CTF immunoreactivities, while the effect of C99^G33L^ on dimerization was low and not significantly different from control cells expressing C99 (Fig. 3b). Indeed, in HEK293 cells, the high expression of C99 was sufficient to induce the presence of HMW APP-CTFs, the levels of which however were found to be increased by D6-treatment (Fig. 3b). Furthermore, as it was the case for C99-expressing cells, mutant (C99^G33L^ and C99^G29L/G33L^) expressing cells also displayed a high level of C83, indicating that these mutations did not alleviate susceptibility to α-secretase cleavage (Fig. 3b, d). Strikingly, the electrophoretic patterns in the mutant expressing cells fully mimicked those observed in D6-treated wild-type C99 expressing cells (Fig. 3b-e). Indeed, in both cases, immunoblots revealed a mix of distinct HMW APP-CTF immunoreactivities, thus reflecting a heterogeneous mix of dimers but also of oligomers composed of both C83 and C99.

### Pharmacological blockade or genetic invalidation of γ-secretase unravels a distinct subcellular localization of monomeric and oligomeric APP-CTFs

**Fig. 4:**
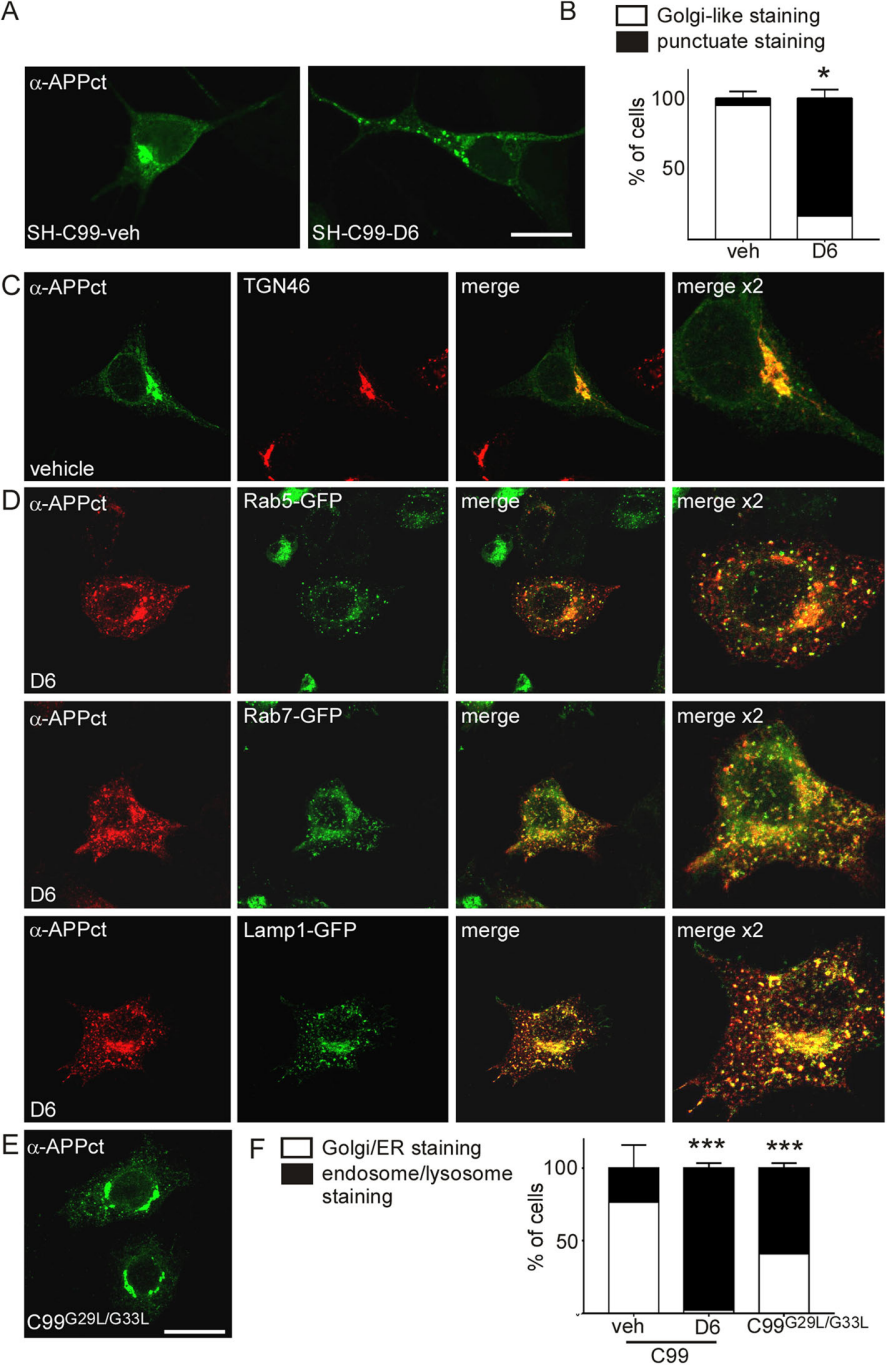
The subcellular localization of APP-CTFs is different in vehicle- and D6-treated C99-expressing SH-SY5Y and HeLa cells*.* **a**-**b** SH-SY5Y cells stably expressing C99 (SH-C99) were treated with or without D6 and immunostained with α-APPct. **a** APP-CTF immunostaining revealed a strong perinuclear staining typical of the Golgi in vehicle-treated cells and a punctuate staining in D6-treated cells. Bars in **b** correspond to the quantification of cells having either Golgi-like or punctuate APP-CTF immunostaining. A total of 400 cells for each condition from 3 independent experiments were counted. Data represent means ± SEM, *p < 0.05 as analyzed by the Mann Whitney U-test. **c**-**f** HeLa cells were transiently transfected with C99 and treated or not with D6 (**c**, **d** and **f**) or with C99^G29L/G33L^ (**e**, **f**). **c**, Vehicle-treated C99-expressing cells presented a strong degree of colocalization of C99 (green) and the *trans*-Golgi apparatus marker TGN46 (red). **d** In D6-treated cells, the APP-CTF immunostaining (red) was punctiform and there was a high degree of colocalization with the subcellular markers Rab5-GFP, Rab7-GFP or Lamp1-GFP (green). Right panels show 2-fold high magnification images. **e** Cells transfected with C99^G29L/G33L^ and APP-CTFs stained using α-APPct displayed a mix of Golgi-like and punctuate staining. **f** Bars correspond to the quantification of cells having either Golgi/ER or endosome/lysosome associated APP-CTF immunostaining. A total of 500 cells for each condition from 3 independent experiments were counted. Data represent means ± SEM, ***p < 0.001 as analyzed by 2-way ANOVA followed by Dunetts posthoc analysis

**Fig. 5:**
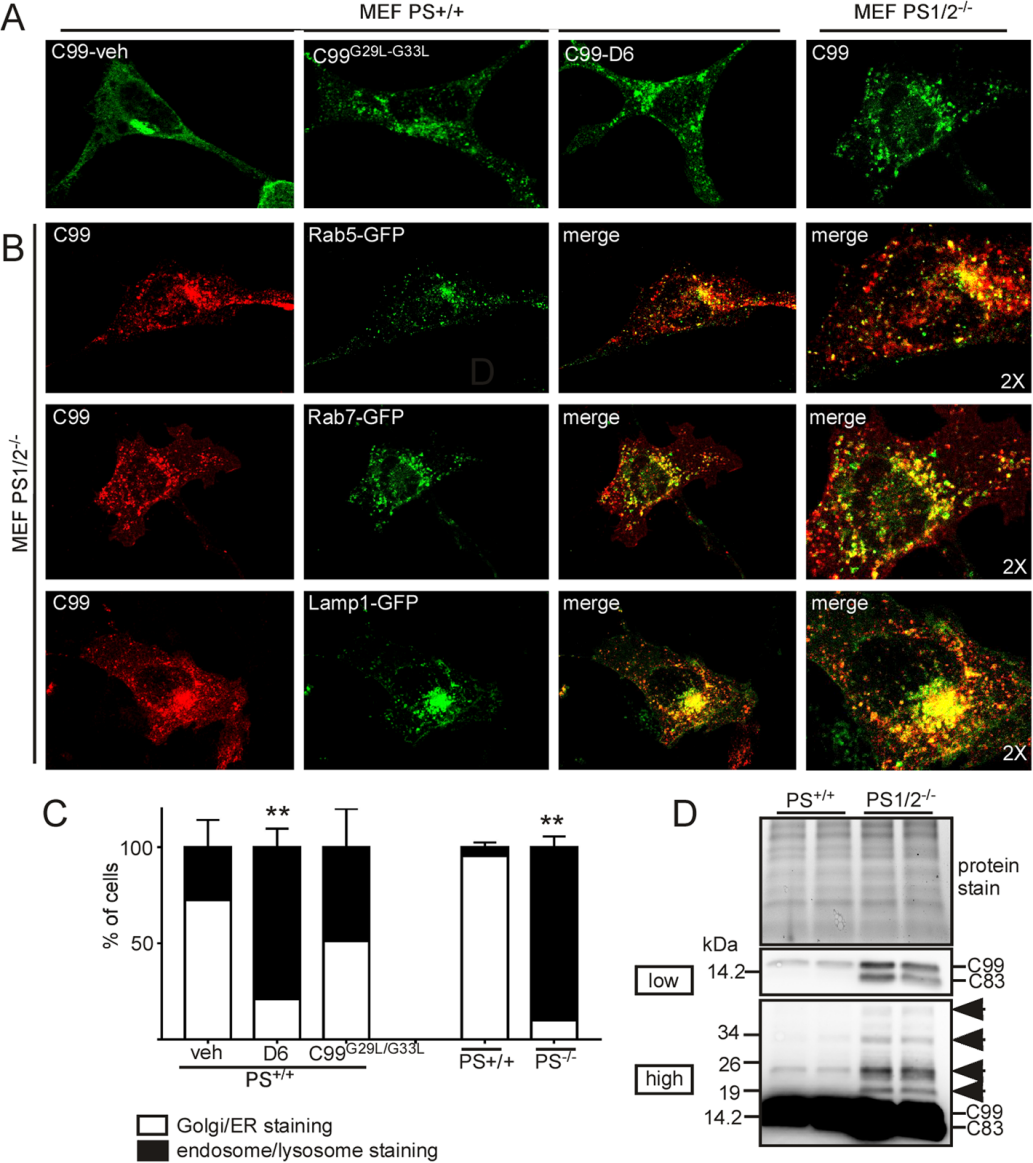
The subcellular localization of APP-CTFs is different in PS^+/+^ and PS1/2^−/−^ MEF cells*.* **a** MEF PS^+/+^ cells transfected with C99 or C99^G29L/G33L^ and treated or not with D6, or MEF PS1/2^−/−^ cells were stained with α-APPct. Whereas vehicle-treated C99-transfected MEF PS^+/+^ cells presented Golgi-like staining, D6-treated cells, aswell as C99^G29L/G33L^ expressing and PS1/2^−/−^ cells, displayed mainly punctuate staining. **b** MEF PS1/2^−/−^ cells were co-transfected with C99 and the intracellular markers Rab5-GFP, Rab7-GFP or Lamp1-GFP (green) and immunostained with α-APPct (red). The right panels show 2-fold high magnification of merged images. **c** Bars correspond to the quantification of cells having either Golgi/ER or endosome/lysosome associated APP-CTF immunostaining. A total of 400 cells for each condition from 3 independent experiments were counted. Data represent means ± SEM, ***p < 0.001 as analyzed by 2-way ANOVA followed by Dunetts posthoc analysis. **d** Immunoblot analysis of APP-CTFs in MEF PS^+/+^ and PS1/2^−/−^ cells. Proteins were stained by photoactivation using the Bio-Rad prestain method (protein stain) and immunoblots used for the detection of APP-CTFs. Arrows point to HMW immunoreactivities

Our data showing a selective enrichment of HMW APP-CTFs in EVs could originate from an initially distinct subcellular localization of monomeric APP-CTFs. Indeed, immunofluorescence analysis of the SH-C99 cells used for EV purifications revealed a distinct APP-CTF immunostaining in vehicle and D6-treated cells (Fig. 4a-b). In vehicle conditions (where almost all CTF species analysed biochemically correspond to monomeric C99 and C83, see Fig. 1), nearly all cells displayed a strong APP-CTF staining localized to the perinuclear region typical of the Golgi apparatus and to the ER (Fig. 4a, b). However, in D6-treated SH-C99 cells (displaying monomeric but also oligomeric APP-CTFs), the immunostaining appeared strongly punctuate in almost all cells (Fig. 4a**,** b). Since SH-5YSY cells, because of their small size, are not very appropriate for subcellular analysis, we decided to use the larger HeLa cells to more accurately delineate the respective intracellular localization of APP-CTF monomers and oligomers (Fig. 4c-f). Also in untreated C99 expressing HeLA cells, the APP-CTF staining was seen within the perinuclear region typical of the Golgi apparatus and found to co-localize with the *trans*-Golgi apparatus marker, TGN46 (Fig. 4c). Conversely, in D6-treated cells there was an almost complete quantitative shift from cells displaying mostly “Golgi/ER” associated staining to those displaying mostly punctuate staining (Fig. 4d, left panel, Fig. 4f). In these cells, there was a strong overlap with markers of early endosomes (Rab5-GFP), late endosomes (Rab7-GFP) as well as lysosomes (Lamp1-GFP) (Fig. 4d) indicating that the punctuate staining corresponded to a mixture of organelles of the endosomal network. Overall, this data suggested that monomers and HMW APP-CTFs have a distinct subcellular localization, with monomers mainly localized to the Golgi and ER and HMW species to endosomes and lysosomes. In agreement, cells transfected with the GXXXG mutant C99^G29L-G33L^ displayed a mix of cells with endosomal/lysosomal-associated staining and Golgi/ER associated staining even in absence of D6 and many cells displayed both Golgi and endolysosomal staining (Fig. 4 e-f). To validate that the D6-associated modulation of APP-CTF localization was genuinely linked to the blockade γ-secretase activity, we performed similar experiments in presenilin 1 and 2 invalidated cells (MEF PS1/2^−/−^), known to totally lack γ-secretase activity [23] (Fig. 5). Firstly, we confirmed a Golgi/ER associated APP-CTF staining in about 95% of C99 transfected MEF-PS-WT cells (Fig. 5a, c) and observed that these cells, when treated with D6 or transfected with C99^G29L/G33L^, displayed a more punctuate APP-CTF immunostaining and a reduced staining in the Golgi (punctuate in about 80 and 50% of D6-treated and C99^G29L/G33L^ transfected cells, respectively) (Fig. 5a, c). Next, we found that about 90% of C99 transfected MEF PS1/2^−/−^ cells displayed a punctuate staining and no or very low Golgi associated staining (Fig. 5a, c). Again, co-labeling with organelle markers revealed the presence of APP-CTF-associated immunoreactivity in early (Rab5-GFP) and late (Rab7-GFP) endosomes, as well as in lysosomes (Lamp1-GFP) (Fig. 5b). Accordingly, immunoblot revealed the higher levels of HMW APP-CTFs in C99 transfected MEF PS1/2^−/−^ as compared to C99-expressing MEF PS^+/+^ cells (Fig. 5d). This data showed that both pharmacological blockade and genetic depletion of γ-secretase yield HMW APP-CTFs species that are selectively associated to endosomal-lysosomal compartments.

### D6-treatment reduces APP-CTF/SorLA physical interaction

**Fig. 6:**
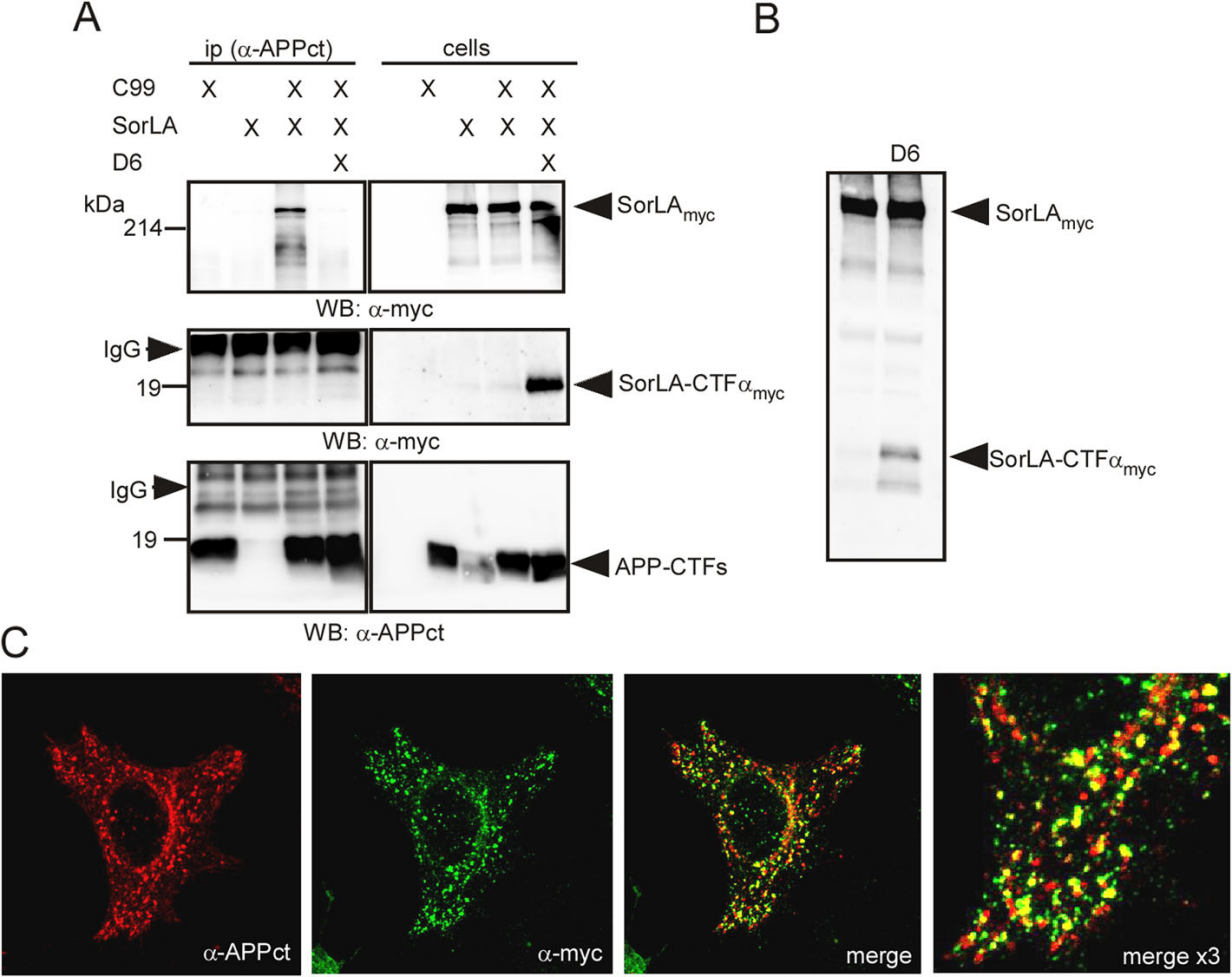
D6-treatment abolishes APP-CTF/SorLA interaction and leads to SorLA-CTFα accumulation in the same intracellular compartments than APP-CTFs. **a** HEK-293 cells stably expressing C99 were transiently transfected with myc-tagged SorlA and treated or not with D6. SorLA was co-immunoprecipitated with α-APP-CTF antibodies only in vehicle treated cells (upper blot). The middle blot corresponds to the lower part of the immunoblot at higher exposure and reveals the presence of SorLA-CTFα in D6-treated cells. The lower blot corresponds to the same protein samples, but blotted with α-APPct, thus showing the levels of immunoprecipitated APP-CTFs. **b** Immunoblot analysis of SorLAmyc in cell lysates of vehicle- or D6-treated HEK-C99 cells revealed the presence of SorLA-CTFα-myc in D6-treated cells. **c** Immunocytochemistry of C99 (red) and SorLAmyc (green) in D6-treated co-transfected HeLA cells

Our data suggest that γ-secretase inhibition leads to APP-CTF oligomers formation by preventing C99 cleavage, thereby increasing the concentrations of APP-CTFs. This cleavage is thought to mainly occur within compartments of the endosomal network suggesting that oligomer formation and accumulation take place within these compartments. However it is also possible that a defective APP-CTF trafficking could contribute to this endolysosomal build-up of oligomeric APP-CTFs. Previous work demonstrated that C99, as it is the case for APP, can bind to the sorting receptor SorLA [31], the major regulator of subcellular shuttling of APP between the TGN and endosomes [32, 33]. Thus, we hypothesized that a defective interaction between SorLA and APP-CTFs and thus altered APP-CTFs trafficking could contribute to the accumulation of oligomers in the endosomal network. Indeed, co-immunoprecipitation experiments confirmed: 1) the ability of SorLA to physically interact with APP-CTF monomers, since SorLA was co-immunoprecipitated with α-APPct antibodies in control conditions (vehicle C99-expressing cells) (Fig. 6 upper panel); 2) a full abolishment of SorLA co-immunoprecipitation in D6-treated cells (Fig. 6a upper panel). This set of data suggests that the physical interaction of APP-CTFs with SorLA could be hampered by APP-CTF oligomerization, thus explaining the D6-associated defect of retrograde transport to the TGN leading to APP-CTF accumulation in all compartments of the endosomal network including lysosomes and exosomes. This lack of interaction could be due to a lack of APP-CTF oligomers to bind SorLA. However, it is also possible that γ-secretase inhibition affects SorLA and thus its ability to bind APP-CTFs, since SorLA is also a well known substrate of γ-secretase [34, 35]. Indeed, SorLA undergoes regulated intramembrane proteolysis with a TACE1-mediated primary cleavage of SorlA yielding a membrane-attached fragment that behaves as a substrate of γ-secretase cleavage, referred to as SorLA-CTFα [34, 35]. This was confirmed in our study, since a low molecular weight immunoreactivity most likely corresponding to SorLA-CTFα accumulates in D6-treated cells (Fig. 6a right middle panel and Fig. 6b). In agreement, immunocytochemistry confirmed a particular strong overlap of SorLA and APP-CTF immunostainings within endolysosomal compartments in γ-secretase inhibitory conditions (Fig. 6c). Thus, our data suggest that a defective γ-secretase cleavage of both APP-CTF and SorLA-CTFα could contribute to the accumulation of oligomeric APP-CTFs.

## Discussion

Our previous work on the 3xTgAD and AAV-C99 mouse models showed that C99 accumulation was both a cause and consequence of early lysosomal dysfunction [7]. We found that the treatment of these animals with the γ-secretase inhibitor D6 led to further increased endolysosomal-associated APP-CTFs and to exacerbated lysosomal-autophagic dysfunction [7]. Here we demonstrate that γ-secretase inhibition also drastically increases exosomal APP-CTF levels reflected by a diffuse extracellular APP-CTF-associated immunostaining in C99-accumulating brain areas, an anatomical observation that was supported by further biochemical analysis. First, we confirmed the presence of both C99 and C83 in purified EVs from both C99- and APPswe-expressing cell media and mouse brains, in agreement with previous studies using purified EVs from cell media [17, 18, 36, 37] or brain tissue from Tg2575 mice [25]. In our models, most C83 was found to derive from α-secretase-mediated cleavage of C99, since the treatment with the α-secretase inhibitor GI254023X led to strongly decreased C83 levels and concomitantly increased C99 levels. In agreement with previous works showing the presence of the α-secretase ADAM10 in EVs [36, 38], this cleavage was found to take place at least partly within exosomes, in which the C83/C99 ratio was even higher than the same intracellular ratio. More surprisingly, purified EVs from γ-secretase inhibitor-treated mice or cell media not only displayed further increased C99 and C83 levels but also HMW APP-CTF immunoreactivities, the levels of which were very low in whole cell or brain homogenates. These HMW APP-CTFs were found in cells expressing C99, but not in control cells, indicating that they were directly linked to C99 and not to other APP-cleavage products, such as the recently discovered CTFƞ [39, 40] proposed to be present in exosomes [37]. Indeed, several lines of independent data from pharmacological (α-secretase inhibition), immunological (C- and N-terminal directed and aggregate specific- antibodies) or genetic (GXXXG mutants and presenilin invalidation) approaches indicated that these HMW APP-CTFs correspond to a heterogenous mix of oligomeric APP-CTFs composed of C99 and C83 homo- and hetero-oligomers. Indeed, in agreement with a selective enrichment of HMW APP-CTFs in exosomes, the treatment with the exosomal inhibitor GW4869 only slightly affected the intracellular levels of monomers, but triggered a significant retention of HMW APP-CTFs within cells. Furthermore, our immunocytochemical analysis indicated that the selective recovery of oligomers in exosomes was linked to their mislocalization within the endolysosomal network. Indeed, we found that monomers were localized mainly in the TGN and ER, whereas oligomers were present in endosomal and lysosomal compartments. It is well accepted that γ-secretase cleavage mainly occurs within acidic compartments of the endocytic pathway [12, 13]. Thus, γ-secretase inhibition-induced oligomer formation most likely takes place within these compartments explaining their specific accumulation in endosomes, lysosomes as well as exosomes. However, in addition our data suggested that this endolysosomal build-up was amplified due to defects in APP-CTF trafficking in γ-secretase inhibitor conditions. Both APP [41] and APP-CTFs [31] are trafficked between the TGN and endosomes by means of sorting molecules including SorLA (sortilin receptor with A-type repeats). This trafficking is mediated via the C-terminal tail of SorLA and its binding to cytosolic adaptor complexes, the retromer (for retrograde transport) and Golgi-localized γ-ear-containing ARF-binding proteins (GGAs) and AP1 and PACS1 (for anterograde transport) [41]. On one hand, the disruption of retromer binding results in a retrograde-sorting defect with accumulation of SorLA and thus APP in endosomes and their depletion from the TGN. On the other hand, the disruption of the GGA interaction increases the levels of SorLA, and thus APP, in the TGN [42]. Here, we showed that D6-treatment led to a strongly reduced SorLA/APP-CTF interaction suggesting that a defective retrograde transport of APP-CTF oligomers could contribute to the endosomal HMW APP-CTF accumulation. Accordingly, a recent paper showed that hippocampal neurons from mice expressing the APP mutant Y682G APP^YG/YG^, that poorly interacts with SorLA, display strong APP accumulation within late endosomes (LEs) and lysosomes, ensuing functional alterations of the lysosomal system [43]. Here, we also found a strong lysosomal accumulation of APP-CTFs in D6-treated conditions, and previously showed that this APP-CTF accumulation could be a trigger of lysosomal and autophagic impairments [7]. Our data suggest that defects in APP-CTF trafficking is due to the fact that oligomeric APP-CTFs poorly bind SorLA. However, it should be noted that SorLA itself undergoes regulated intramembrane proteolysis (RIP) with an initial ectodomain shedding by α-secretase yielding SorLA-CTFα that undergoes subsequent γ-secretase-mediated hydrolysis generating SorLA-CTFγ (SorLA-ICD) [34, 35]. Indeed, D6 treatment led to the accumulation of SorLA-CTFα within the same intracellular compartments as APP-CTF oligomers suggesting that endosomal accumulation of SorLA-CTFα *perse* could affect SorLA function and APP-CTF trafficking. Whatever the exact mechanisms, our findings indicate that γ-secretase activity is critical for a correct APP-CTF trafficking, since the phenotype elicited by the pharmacological blockade of γ-secretase was strictly mimicked by the genetic depletion of presenilins that correspond to the catalytic core of the enzymatic complex [44, 45].

Previous in vitro data have indicated that C99 can dimerize [19], but our work is the first to demonstrate that APP-CTFs can also heterodimerize and form oligomers and to show the presence of oligomeric APP-CTFs in vivo in AD mouse models. We found that the GXXXG mutant C99^G29L/G33L^ mimics the γ-secretase inhibitor and blocks oligomeric APP-CTFs within compartments of the endosomal network. Probably a modified conformation of this mutant makes it more prone for dimerization, but the fact that dimers selectively accumulate in endosomes suggest that dimerization takes place within these compartments and indicates that oligomerization requires a particular biological environment (membrane lipid composition, pH etc). Indeed, previous works have shown that C99 dimerization is strongly affected by changes in both membrane thickness and lipid composition [46]. Thus taken together, our data indicate that APP-CTF oligomerization depends on the structural conformation of C99, the levels of γ-secretase activity and the intracellular localization, and propose that the targeting of APP-CTF oligomers to late endosomes, lysosomes and exosomes is also linked to a defective APP-CTF retrograde transport to the TGN. Oligomer misdistribution may well contribute to the previously described endolysosomal defects in mouse AD models [7, 8], as well as in human AD affected brains [47] or primary fibroblasts from individuals with Down Syndrome [9, 48]. Although our studies are based on overexpression models, the conclusions concernant endolysosomal accumulation of aggregated C99 are strengthened by recent works based on either isogenic knockin human iPSCs modified by CRISPR/Cas9 or monogenic iPSCs carrying either APP or PS1 mutations, and which both demonstrate C99-mediated and Aβ-independent alterations of the endolysosomal network [49]. Whereas the paper of Kwart observed early endosomal dysfunction, the work from Hung and Livesey demonstrated lysosomal-autophagic pathology [6]. Similar findings were described by the group of Dr. Nixon using fibroblasts from Down’s patients known to display an elevation of C99. These cells were also found to display defects in early endososomes [4, 50] as well as in lysosomal degradation [9]. Again, these alterations were found to be mediated by C99 and to be Aβ independent.

Interestingly, the data obtained in our study reconcile our immunohistochemical studies describing two distinct conformation- and localization-dependent APP-CTFs in our mouse models. Thus, in situ*,* only monomeric APP-CTFs localizing to the TGN and the ER, are recognized by C-terminal directed antibodies, whereas oligomerized and monomeric but oligomerization-prone APP-CTFs present in endosomes, lysosomes and exosomes, are detected with aggregate-specific antibodies but not with C-terminal antibodies.

## Conclusions

**Fig. 7:**
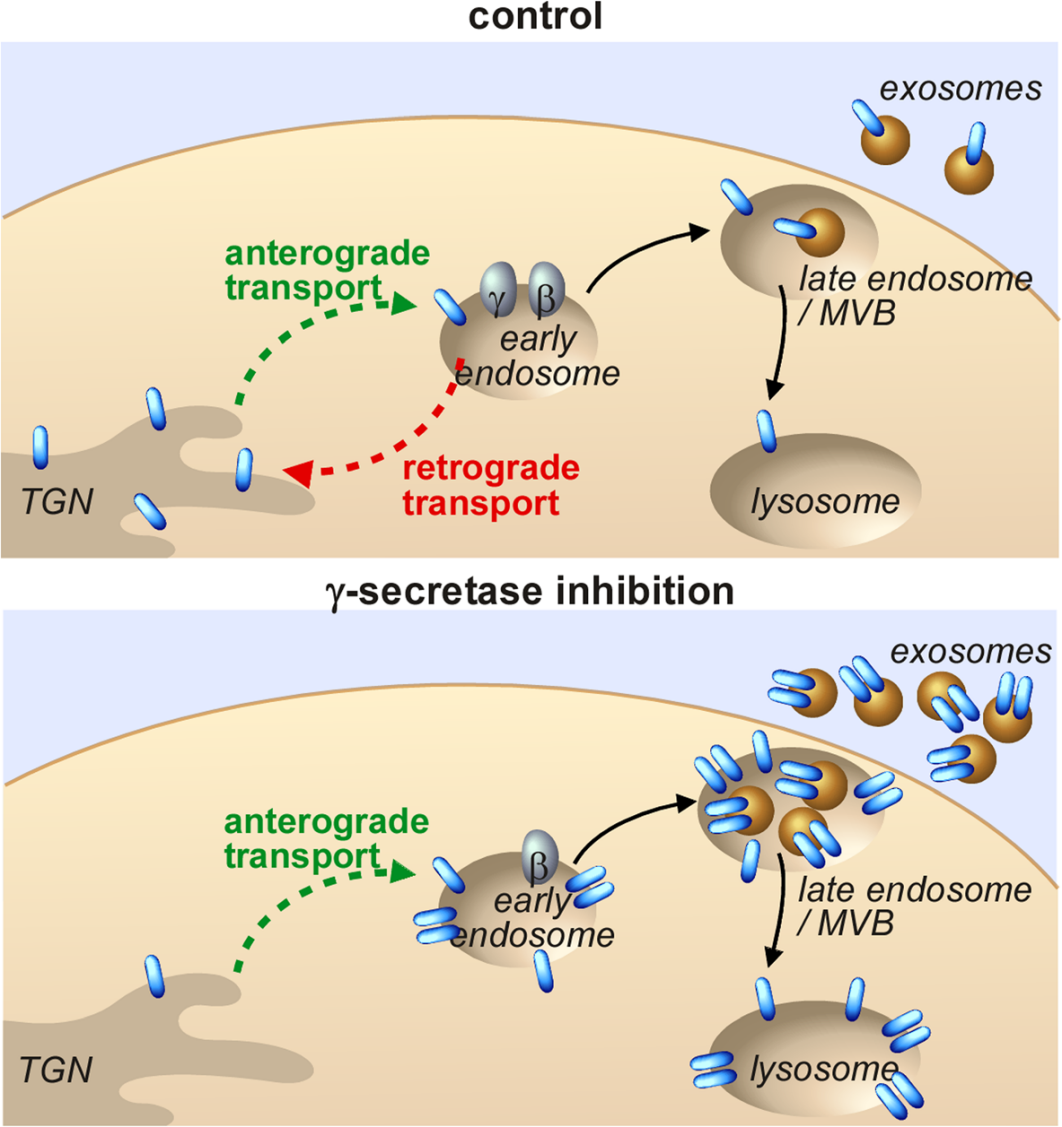
Hypothetical model of oligomeric APP-CTF-enrichment in exosomes after γ-secretase inhibition. In control conditions, C99 (blue) monomers are trafficked between the TGN and endosomes and mainly accumulate in the Golgi. However, the inhibition of γ-secretase triggers the formation of higher levels of oligomeric APP-CTFs within endosomes, the main sites of γ-secretase cleavage. These oligomers poorly bind to SorLA, thus leading to a defect in their retrograde trafficking to the TGN and misdistribution to late endosomes, lysosomes as well as to the endosome-originating exosomes. γ and β corresponds to γ-secretase and β-secretase, respectively

This study is the first to demonstrate the presence of oligomeric APP-CTFs in in vivo AD mouse models and to show that their levels are strongly increased upon γ-secretase inhibition. We found that, unlike monomeric C99 that is mainly localized to the TGN, oligomeric APP-CTFs are mislocalized to compartments of the endosomal-lysosomal network including the endosomal-originating exosomes (see hypothetical model in Fig. 7). Our findings open a novel view of APP-CTF related toxicity linked to their propensity to oligomerise and potentially spread via exosomes. The specific contribution of these oligomeric APP-CTF-bearing exosomes to AD pathology remain to be established. Of note, our previous work unveiled pronounced C99-associated inflammatory stigmata in our mouse models [7]. Interestingly, we here found that a subset of neuron-derived EVs are taken up by microglia, the immune cells of the brain, providing an anatomical support to explain this neuroinflammation, although the definitive contribution of exosomal APP-CTFs in this process remains to be established. Indeed, microglial uptake of pathogenic EVs could have several consequences. On the one hand, it can serve as a means for the clearance of toxic molecules, as it was proposed for Aβ [51]. On the other hand, microglial uptake can contribute to disease propagation by spreading toxic molecules, as proposed for hyperphosphorylated tau [52], and can also initiate neuroinflammation [53]. Future studies should allow determining the exact role of these APP-CTF bearing EVs in neuron to neuron and neuron to glial cell propagation and thus AD disease progression. Finally, our findings indicating that γ-secretase inhibition amplifies the presence of EVs carrying particular APP-CTFs adds a new argument against a therapeutic strategy based on γ-secretase inhibition in AD. According to our observations, these inhibitors should amplify the load and exosomal spread of toxic APP-CTF species.

## Data Availability

The datasets used and/or analysed during the current study available from the corresponding author on reasonable request.

## Abbreviations

AAV: adenoassociated virus
AD: Alzheimer’s disease
APP: amyloid precursor protein
CTF: C-terminal fragment
DAB: 3,3-diamonobenzidine
EM: electron microscopy
ER: endoplamic reticulum
EV: extracellular vesicle
Gi: a-secretase inhibitor GI254023X
GW: exosomal inhibitor GW4869
HEK: human embryonay kidney
HeLA: human epitheloid cervix carcinoma
HMW: high molecular weight
MEF: mouse embryonary fibroblasts
MVB: multivesicular body
nSMAse: neutral spingomyelinase
NTA: nanoparticle tracking analysis
PBS: phosphate buffered saline
PFA: paraformaldehyde
PS: presenilin
SH-C99: C99 expressing SH-SY5Y neuroblastoma
SorLA: sortilin receptor with A-type repeats
swe: Swedish mutation
Tg: transgenic
TGN: trans-Golgi network
WT: wild-type
